# Characterization of tumoricidal activities mediated by a novel immune cell regimen composing interferon-producing killer dendritic cells and tumor-specific cytotoxic T lymphocytes

**DOI:** 10.1186/s12885-024-12101-3

**Published:** 2024-03-28

**Authors:** Chih-Hao Fang, Wen-Fang Cheng, Ya-Fang Cheng, Keng-Li Lan, Jan-Mou Lee

**Affiliations:** 1 Biomedical Industry Ph.D. Program, College of Life Sciences, National Yang Ming Chiao Tung University, Taipei, Taiwan; 2 FullHope Biomedical Co.,Ltd, 10F., No. 10, Ln. 609, Sec. 5, Chongxin Rd., Sanchong Dist., New Taipei City, 241405 Taiwan; 3 Graduate Institute of Clinical Medicine, College of Medicine, National Taiwan University, Taipei, Taiwan; 4 Department of Obstetrics and Gynecology, College of Medicine, National Taiwan University, Taipei, Taiwan; 5 Graduate Institute of Oncology, College of Medicine, National Taiwan University, Taipei, Taiwan; 6 Department of Heavy Particles & Radiation Oncology, Taipei Veterans General Hospital, Taipei, Taiwan; 7 Institute of Traditional Medicine, School of Medicine, National Yang Ming Chiao Tung University, No. 155, Sec. 2, Linong St. Beitou Dist., Taipei City, 112304 Taiwan

**Keywords:** Phyduxon-T, IKDCs, TAA-specific CD8 T cells, 4-1BB, Immunotherapy

## Abstract

**Background:**

Although immune cell therapy has long been used for treating solid cancer, its efficacy remains limited. Interferon (IFN)-producing killer dendritic cells (IKDCs) exhibit cytotoxicity and present antigens to relevant cells; thus, they can selectively induce tumor-associated antigen (TAA)-specific CD8 T cells and may be useful in cancer treatment. Various protocols have been used to amplify human IKDCs from peripheral sources, but the complexity of the process has prevented their widespread clinical application. Additionally, the induction of TAA-specific CD8 T cells through the adoptive transfer of IKDCs to immunocompromised patients with cancer may be insufficient. Therefore, we developed a method for generating an immune cell-based regimen, Phyduxon-T, comprising a human IKDC counterpart (Phyduxon) and expanded TAA-specific CD8 T cells.

**Methods:**

Peripheral blood mononuclear cells from ovarian cancer patients were cultured with human interleukin (hIL)-15, hIL-12, and hIL-18 to generate Phyduxon-T. Then, its phenotype, cytotoxicity, and antigen-presenting function were evaluated through flow cytometry using specific monoclonal antibodies.

**Results:**

Phyduxon exhibited the characteristics of both natural killer and dendritic cells. This regimen also exhibited cytotoxicity against primary ovarian cancer cells and presented TAAs, thereby inducing TAA-specific CD8 T cells, as evidenced by the expression of 4-1BB and IFN-γ. Notably, the Phyduxon-T manufacturing protocol effectively expanded IFN-γ-producing 4-1BB^+^ TAA-specific CD8 T cells from peripheral sources; these cells exhibited cytotoxic activities against ovarian cancer cells.

**Conclusions:**

Phyduxon-T, which is a combination of natural killer cells, dendritic cells, and TAA-specific CD8 T cells, may enhance the efficacy of cancer immunotherapy.

**Supplementary Information:**

The online version contains supplementary material available at 10.1186/s12885-024-12101-3.

## Introduction

Immune surveillance is essential for resisting pathogen invasion and curbing tumor cell transformation. Innate killer cells, such as natural killer (NK) cells, kill transformed cells by recognizing the aberration or downregulation of major histocompatibility complex (MHC)-I. Subsequently, tumor-associated antigens (TAAs), which are protein debris released from dying tumor cells, are phagocytosed by antigen-presenting cells (APCs), including dendritic cells (DCs). These DCs then migrate to the draining lymph nodes and present TAAs to induce naive T cells to become TAA-specific T cells. Finally, these TAA-specific T cells migrate to tumor sites and execute long-lasting adaptive tumor-specific T-cell responses [1, 2].

Various immune cell-based therapies have been developed to control cancer progression. However, the clinical application of these therapies has encountered challenges. For example, the adoptive transfer of NK cells can effectively drive innate cell-driven tumor destruction. Still, it lacks the APC function required to capture released tumor antigens, leading to an insufficient induction of memory T cell responses [3]. Conversely, DC vaccine-primed T cells or engineered T cells, including chimeric antigen receptor T (CAR-T) cells and T-cell receptor (TCR)-engineered T cells (TCR-T cells), potentially provide long-lasting T-cell immune responses targeting specific tumor antigens. However, the absence of innate killer cell activity has reduced their efficacy against dynamic changes in tumor antigen epitopes during tumor immune escape [4, 5]. Thus, there is an urgent need for an immune cell-based treatment regimen that incorporates innate killer activities to release tumor antigens and APC functions to process the released antigens and generate TAA-specific T cells. Such a regimen would activate TAA-specific CD8 T cells, harnessing the power of comprehensive immune surveillance to overcome tumor immune escape and combat cancer.

Studies using murine models have identified a rare chimeric cell population called interferon (IFN)-producing killer dendritic cells (IKDCs), exhibiting both NK and DC activities. IKDCs can kill tumors, function as APCs by capturing apoptotic tumor cells [6–8], and target and eliminate tumors while cross-presenting tumor antigens to prime CD8 T cells with tumor specificity [9]. In other words, they can exert innate killer cell cytotoxicity and present tumor antigens, bridging adaptive CD8 T-cell immune responses against tumors. The human counterpart of IKDC was initially identified as CD56^+^HLA-DR^+^ [10]. Despite attempts to amplify these rare cells from the periphery using various in vitro expansion protocols, the need for cell depletion during the initial cell preparation stage has limited their clinical applicability [11–13]. Additionally, following the adoptive cell transfer of human IKDCs, the yield and functionality of TAA-specific CD8 T cells may be insufficient [14].

Ovarian cancer (OC) stands out as an immunogenic cancer and a promising target for immunotherapy [15]. Nevertheless, the clinical outcomes of various immunotherapies have proven disappointing [16]. One significant hurdle is tumor immune escape, characterized by OCs lacking or losing tumor-specific antigens, impeding the generation of TAA-specific T cells for effective recognition and attack [17, 18]. In this study, we gathered peripheral blood from OC patients to develop an immune cell-based regimen named Phyduxon-T, combining a novel human IKDC counterpart (Phyduxon, derived from NK cells with acquired DC characteristics and functions) with expanded TAA-specific T cells. Co-culturing Phyduxon with primary OC cells revealed its ability to kill OC cells and present tumor antigens. This process facilitated the selection of TAA-specific CD8 T cells expressing 4-1BB and IFN-γ. Notably, the Phyduxon-T manufacturing process also created a favorable environment for the *in-vitro* expansion of 4-1BB^+^ TAA-specific CD8 T cells producing IFN-γ, thereby enhancing T cell-mediated tumoricidal activity. Hence, our immune cell-based regimen, integrating NK cells, DCs, and TAA-specific CD8 T cell functions, represents a novel and promising approach to cancer treatment.

## Materials and methods

### Reagents and antibodies

Detailed information on the reagents and antibodies used in this study is listed in Tables S2 to S5.

### Subject enrollment

This study, conducted in accordance with the Declaration of Helsinki, received approval from the Institutional Review Board (IRB) of the National Taiwan University Hospital (202108121RSD, October 22, 2022). Informed consent was obtained from all participants involved in the study. A total of six patients were enrolled, all with histologically confirmed stage I to IV ovarian cancer or recurrent ovarian cancer (Table S1). Exclusion criteria included severe, uncontrolled clinical conditions affecting the heart, liver, or kidneys, infectious diseases, or CTCAE grade 2 or higher pulmonary or abdominal effusion. Experimental drug treatments were prohibited within the 28 days prior to study enrollment. On the day of surgery, approximately 40 ml of peripheral blood, residual tissue (1 cm x 1 cm x 0.5 cm), and 300–500 ml of ascites were collected from each patient.

### Cell lines and primary tumor cell isolation

The human chronic myelogenous leukemia cell line K562 was purchased from the Bioresource Collection and Research Center (Taiwan, 60,007). K562 cells were cultured in Iscove’s Modified Dulbecco’s Medium (IMDM) supplemented with 10% fetal bovine serum (FBS) and passaged every 2 or 3 days. K562 cells were tested to ensure the absence of mycoplasma contamination.

OC cells were isolated from tissue or ascites [19, 20]. Briefly, tumor tissues were minced into small pieces and digested with a mixture of 0.05 ng/mL collagenase type IV, 0.01 ng/mL DNase I, and 0.025 ng/mL hyaluronidase for 30 min at 37 °C with 5% CO_2_ atmospheres. The digested solution was passed through a 100 μm cell strainer and resuspended in a 1:1 mixture of Medium 199 and MCDB (Molecular Cellular Developmental Biology) 105 Medium. Ascites were washed twice at 400 × *g* for 5 min with 1X phosphate-buffered saline (PBS). Mononuclear cells were isolated through Ficoll-Hypaque density gradient centrifugation at 930 × *g* for 30 min at room temperature. The isolated tumor cells were resuspended in a mixture of 50% Medium 199 and 50% MCDB 105 medium and half autologous ascitic fluid for further cultivation.

### PBMC isolation and cell enrichment

Peripheral blood collected from patients with OC was diluted with an equal volume of 1X PBS. Ficoll-Hypaque was slowly loaded into a cell fraction and subjected to density gradient centrifugation at 930 × *g* for 30 min at room temperature. PBMCs were then collected from the buffy coat layer. Magnetic-activated cell sorting (MACS cell) separation was used for cell enrichment (Table S3). For preparing CD25 responder T cells, PBMCs were labeled with biotinylated anti-CD25. For preparing CD8 T cells, PBMCs were labeled with biotinylated anti-CD56, anti-CD4, anti-CD11c, anti-CD14, and anti-CD19. For preparing pure Phyduxon and T cells, Phyduxon-T was labeled with biotinylated anti-CD3 and anti-CD56, respectively. All cells were labeled with biotinylated antibodies for 8 min at room temperature. Biotin-conjugated cells were labeled with streptavidin (SA) microbeads for 10 min at room temperature. The labeled cells were passed through LD columns under a strong magnetic field and collected from the flow-through and eluate fractions.

### Preparation of Phyduxon-T

The Phyduxon-T manufacturing process was modified from the previous study [13]. Briefly, PBMCs were cultured in AIM-V medium with 4% human platelet lysate (HPL), 30 ng/mL human interleukin (hIL)-15, 3 ng/mL hIL-12, and 50 ng/mL hIL-18 at 37 °C under 5% CO_2_. On day 3, the cells were subcultured in AIM-V medium with 4% HPL, 30 ng/mL hIL-15, 3 ng/mL hIL-12, and 50 ng/mL hIL-18. On day 6, 50% of the cells were harvested and pelleted through centrifugation (at 400 ×*g* for 5 min); the cell pellets were resuspended in AIM-V medium containing 4% HPL, 30 ng/mL hIL-15, and 3 ng/mL hIL-12. On day 9, the remaining 50% of the cells were subjected to an identical process. Phyduxon-T was harvested on day 12 to evaluate its phenotype and functional activities.

### Flow cytometry

Flow cytometry was used to assess the phenotype of Phyduxon-T. Immune profiling for Phyduxon and T cells involved staining with specific monoclonal antibodies and corresponding isotype controls (Table S4). Immune profiling for cytotoxic molecules included cell stimulation using Cell Stimulation Cocktail (a cocktail of phorbol 12-myristate 13-acetate (PMA) and ionomycin) and brefeldin A for 2 h at 37 °C with 5% CO2 atmospheres. The cells were stained with the monoclonal antibodies and corresponding isotype controls listed in Table S5 for 10 min at 4 °C. After 20 min of fixation and permeabilization at 4 °C, intracellular staining was performed using specific monoclonal antibodies and corresponding isotype controls listed in Table S5 for 30 min at 4 °C. Samples were acquired using a Navios flow cytometer (Beckman Coulter), and all related data were analyzed with Kaluza (Beckman Coulter, version 2.1).

### Cytotoxic assay

The cytotoxicity of Phyduxon was evaluated using the PanToxiLux kit by assessing granzyme B activity and caspase activation in tumor cells [21]. K562 cells and primary OC cells were stained with TFL4 for 50 min. On day 12, total Phyduxon-T, sorted Phyduxon or sorted T cells out of total Phyduxon-T were incubated with TFL4^+^ K562 cells (ratio of effector to target cells, 5:1) for 20 min and TFL4^+^ OC cells (effector to target cell ratio, 20:1) for 60 min, respectively. The expression level of TFL4^+^ granzyme B/caspase^+^ was evaluated through flow cytometry, and all related data were analyzed using Kaluza.

### APC activity assay

Allogeneic responder cells (CD25^−^ PBMCs) were enriched and stained with the CellTrace CFSE (Carboxyfluorescein succinimidyl ester) cell proliferation kit in the mixed lymphocyte reaction assay. CFSE^+^ cells were cocultured with sorted Phyduxon (ratio of APCs to responder cells, 2:1) for 5 days in the presence of IL-2 and IL-15 to reduce the TCR threshold. The CFSE-diluted pattern of CD3 T cells was evaluated through flow cytometry. To establish a triple-cell coculture model, primary OC cells were cocultured with sort-out Phyduxon (ratio of effector to target cells, 20:1) for 2 days; next, CellTrace Violet-labeled CD8 T cells were added to the target cells and cultured for another 4 days in the presence of IL-2 and IL-15. Violet, 4-1BB, and IFN-γ expression levels on CD8 T cells were evaluated using flow cytometry, and all related data were analyzed using Kaluza.

### Statistical analysis

Data in two-group comparisons were analyzed using the unpaired, nonparametric, two-tailed Mann-Whitney test. Data in three-group comparisons were analyzed using the One-Way ANOVA (Kruskal-Wallis test) and Dunn’s multiple comparisons test for further multiple comparisons. Correlations were assessed using Pearson’s r, and regression analysis was performed using simple linear regression. All statistical analyses were conducted using GraphPad Prism (version 9.1.2) for Windows. Results were considered statistically significant at *p* < 0.05.

## Results

### Phyduxon is a novel human IKDC counterpart exhibiting NK cell and DC phenotypes and activities

**Fig. 1:**
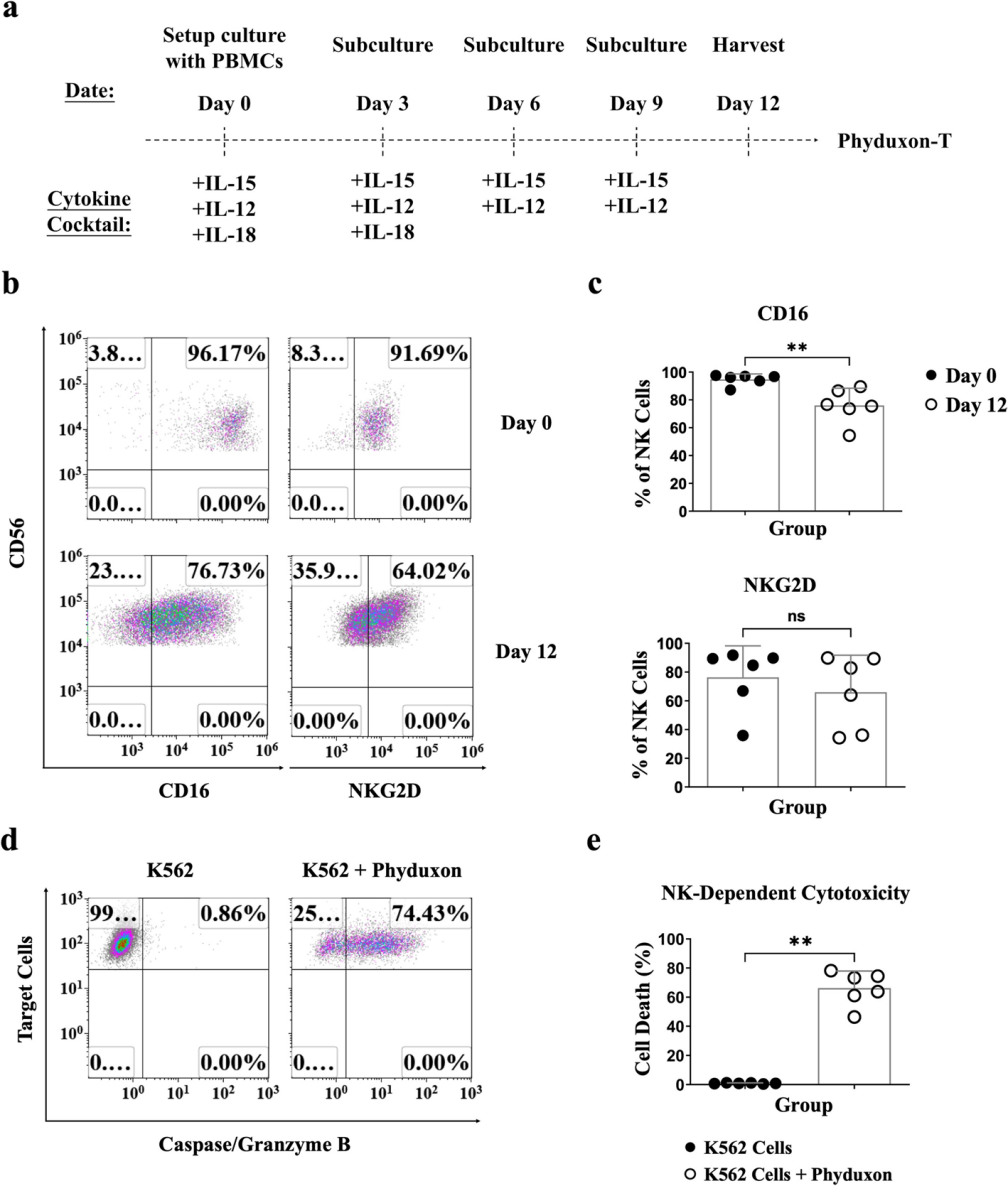
Phyduxon, as a human IKDC counterpart, exhibited the phenotypes and activities of NK cells. **a** Schematic of the manufacturing process for Phyduxon-T. PBMCs were cultured in AIM-V medium plus HPL, containing hIL-15 (days 0, 3, 6, and 9), hIL-12 (days 0, 3, 6, and 9), and hIL-18 (days 0 and 3) for 12 days to generate Phyduxon-T. **b**, **c** Comparison of the expression of CD16 and NKG2D on CD3^−^CD14^−^CD19^−^CD45^+^CD56^+^cells between days 0 and 12 through flow cytometry. **d**, **e** Phyduxon was isolated and evaluated for its cytotoxicity after coculturing with K562 tumor cells, analyzing the TFL-4^+^caspase/granzyme B^+^ pattern through flow cytometry. The results shown in (**b**) and (**d**) are representative of six independent experiments. Data in (**c**) and (**e**) are presented as means ± SD of six independent experiments. Differences between groups were analyzed using the nonparametric Mann-Whitney test. *p *values: **p < 0.01; ns, not significant

We developed a manufacturing platform for Phyduxon-T by building upon our previous study [13]. In this platform, we utilized hIL-15 and hIL-12 to induce the differentiation of initial NK cells, facilitating the acquisition of DC phenotypes and activities. This specialized regimen, termed Phyduxon, was further enhanced with IL-18 to boost the activities of both NK cells and DCs, thereby improving the overall production of Phyduxon (Fig. 1a).

**Fig. 2:**
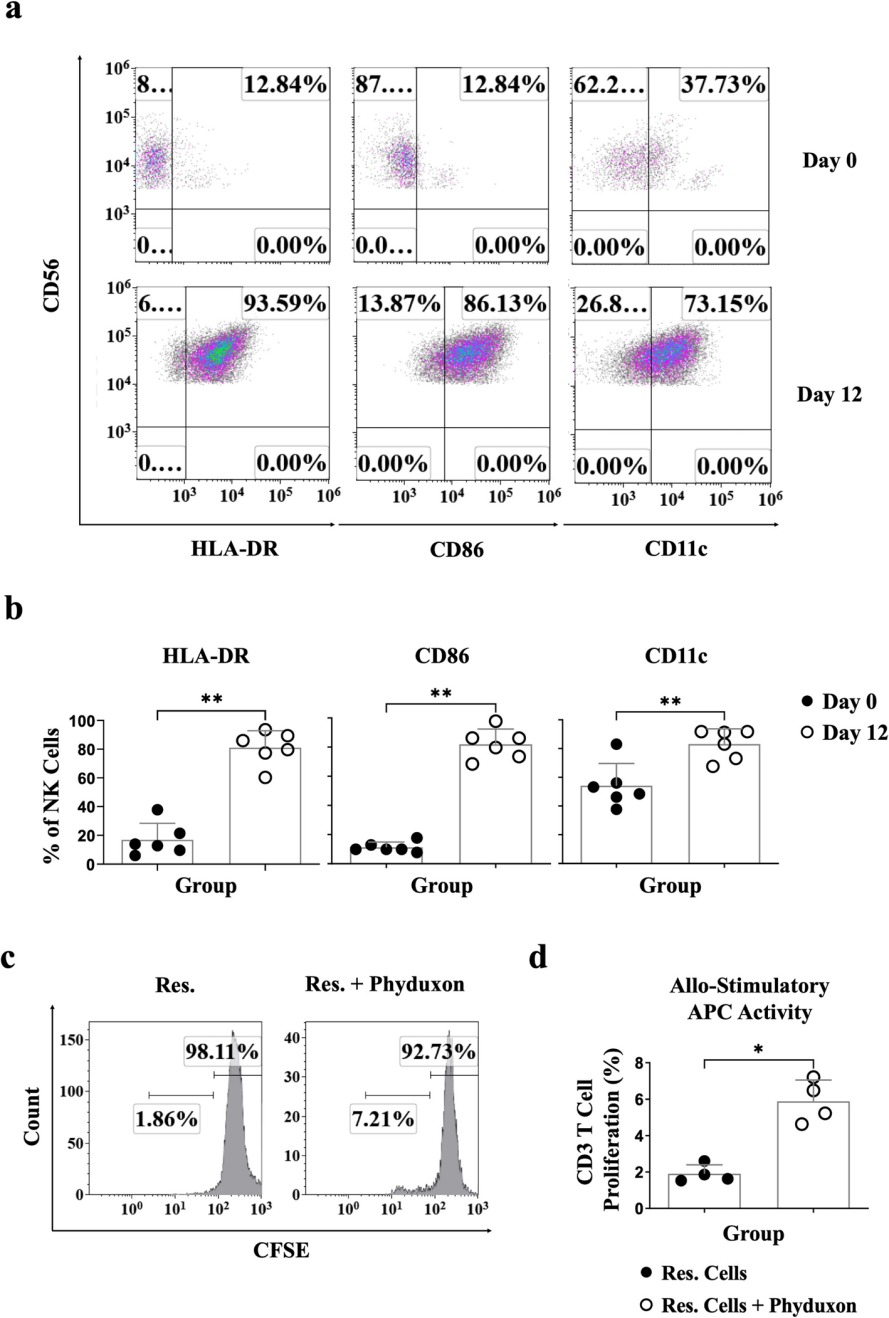
Phyduxon, as a human IKDC counterpart, exhibited the phenotypes and activities of DCs. **a**, **b** Comparison of the expression levels of HLA-DR, CD86, and CD11c on CD3^−^CD14^−^CD19^−^CD45^+^CD56^+^cells between days 0 and 12 through flow cytometry. **c**, **d** Phyduxon was isolated, and its allostimulatory APC function was evaluated by coculturing with allogeneic CFSE-labeled CD25^−^-responder cells (Res.). Responder CD3 T-cell proliferation was assessed by determining the pattern of CFSE dilution through flow cytometry. The results shown in (**a**) and (**c**) are representative of six and four independent experiments, respectively. Data in (**b**) and (**d**) are presented as means ± SD of six and four independent experiments, respectively. Differences between groups were analyzed using the nonparametric Mann-Whitney test. *p *values:*p < 0.05,**p < 0.01

Following a 12-day Phyduxon-T manufacturing process, we conducted the phenotypic analysis to discern NK and DC phenotypes on Lineage-negative (Lin^−^) CD56^+^ cells (Fig. S1). As a result, the Lin^−^CD56^+^ NK cells demonstrated a substantial expression of NK cell phenotype, as evidenced by the expression of CD16 (*p* = 0.0043) and NKG2D (*p* = 0.3939) (Fig. 1b and c and Fig. S1). Furthermore, the sorted CD56^+^ NK cells exhibited cytotoxicity against K562 target cells, with 74.43% showing caspase/granzyme B^+^ characteristics, indicating the presence of NK-dependent cytotoxicity (*p* = 0.0022) (Fig. 1d and e and Fig. S2a). On the other hand, the Lin^−^CD56^+^ NK cells displayed an upregulated expression of DC markers, including HLA-DR (*p* = 0.0022), CD86 (*p* = 0.0022), and CD11c (*p* = 0.0087) (Fig. 2a and b). The sorted CD56^+^ NK cells exhibited an allostimulatory APC function, leading to a 7.21% increase in the proliferation of allogeneic CD3^+^ responder (Res.) T cells (Fig. 2c and d). These findings illustrate that the Phyduxon-T manufacturing process induces the differentiation of CD56^+^ NK cells into Phyduxon, a novel human IKDC counterpart expressing CD3^−^CD14^−^CD19^−^CD45^+^CD56^+^NKG2D^+^CD16^+^HLA-DR^+^CD86^+^CD11c^+^, with both NK cell and DC functions.

### Phyduxon exhibited APC activity by capturing and presenting tumor antigens to T cells, thus inducing TAA-specific CD8 T cells

**Fig. 3:**
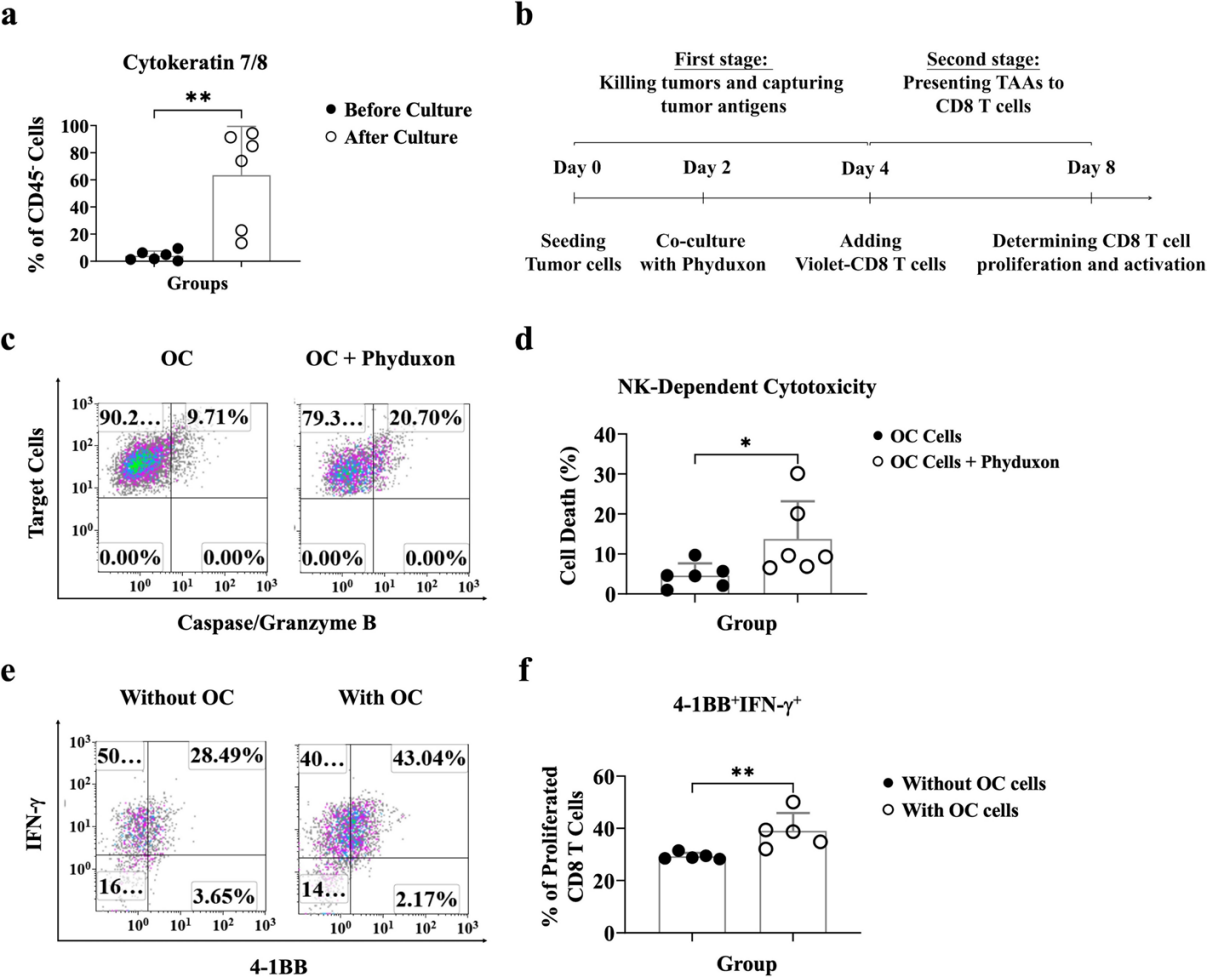
Phyduxon exhibited NK cell-dependent cytotoxicity and induced proliferation and AIM expression of CD8 T cells. **a** Freshly isolated and cultured OC cells were stained with monoclonal antibodies. The expression of cytokeratin-7/8 on CD45^−^ cells was evaluated through flow cytometry. **b** A triple-cell coculture assay was performed to determine the APC function of Phyduxon after engaging with primary OC cells. **c**, **d** OC cells were cocultured with Phyduxon, and the TFL-4^+^caspase/granzyme B^+^ pattern was analyzed through flow cytometry to quantify apoptosis. **e**, **f** Phyduxon was cocultured with or without OC cells to determine the expression levels of IFN-γ and 4-1BB on proliferated CD3^+^CD8^+^ T cells. The results shown in (**c**) and (**e**) are representative of six and five independent experiments, respectively. Data in (**a**) and (**d**) are presented as means ± SD of six independent experiments, while data in (**f**) are presented as means ± SD of five independent experiments. Differences between groups were analyzed using the nonparametric Mann-Whitney test. *p *values: * p < 0.05, ** p < 0.01

Given the potential of Phyduxon in both NK cells and DCs, evaluating its ability to kill tumor cells, capture TAAs, and present TAAs to T cells is crucial. To assess the APC function of Phyduxon in vitro, we developed a primary OC cell culture model. Established OC cells displayed an upregulated expression of cytokeratin-7/8 (*p* = 0.0022), commonly occurring in malignant neoplasms (Fig. 3a) [22, 23].

For the investigation of Phyduxon’s APC function, we measured the proliferation and activation of CD8 T cells using a triple-cell coculture model (Fig. 3b). Phyduxon exhibited NK-dependent cytotoxicity and induced an approximately one-fold increase (*p* = 0.0260) in the apoptosis of OC cells (20.7%) compared to that observed in OC cells cultured alone (9.71%; Fig. 3c, d). To further explore whether TAA-specific T cells can be primed after the Phyduxon-mediated death of OC cells, we utilized CellTrace Violet and activation-induced markers (AIMs), including 4-1BB and IFN-γ, to identify the population of TAA-specific CD8 T cells [24]. Consequently, following the Phyduxon-mediated death of OC cells, we observed an approximately 25% increase (*p* = 0.0079) in the levels of autologous proliferated 4-1BB^+^IFN-γ^+^CD8 T cells compared to those observed without tumor engagement (Fig. 3e and f). These findings indicate that Phyduxon promotes the expansion of tumor-reactive CD8 T cells after engaging with tumors.

### Emergence of AIM^+^ CD8 T cells in the Phyduxon-T manufacturing process

While TAA-specific T cells carrying 4-1BB and IFN-γ can be primed after coculturing with tumor-loaded Phyduxon, the limited yield of TAA-specific T cells may hinder their clinical application (Fig. 3). Thus, strategies for the *in-vitro* expansion of TAA-specific CD8 T cells are crucial. IL-15 plays a role in the differentiation, persistence, and maintenance of T cell memory [25, 26]. Recently, IL-15-encoded CAR-T demonstrated superior memory-like T-cell expansion and persistence, enhancing antitumor effects in various CAR-T pipelines [27]. IL-12 and IL-18, alone with IL-15, enhanced the IFN-γ production of memory CD8 T cells [28]. Therefore, we hypothesized that the cytokine cocktail used in the Phyduxon-T manufacturing protocol might expand a small population of TAA-specific CD8 T cells during Phyduxon generation.

**Fig. 4:**
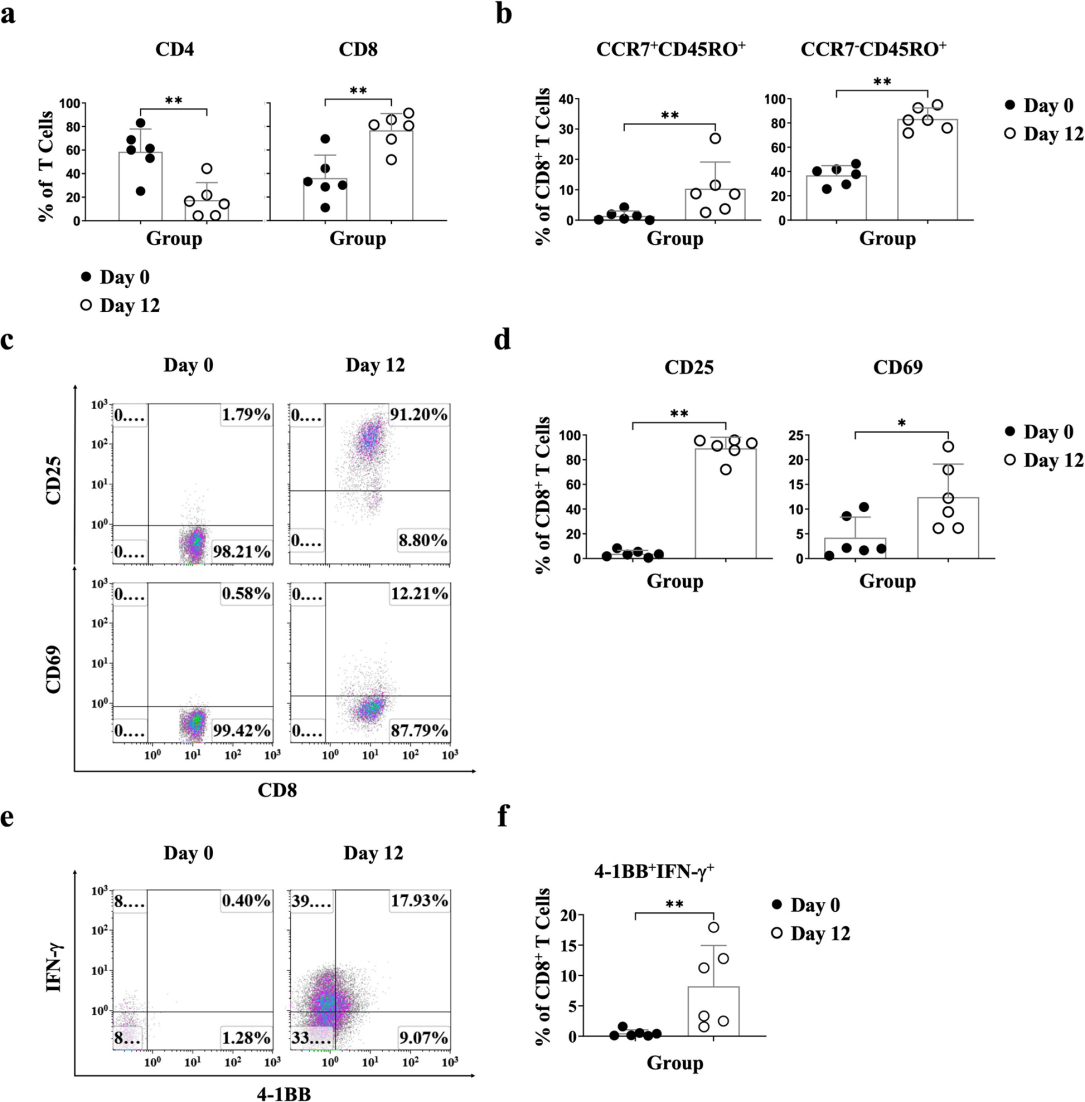
Antigen-exposed CD8 T cells were selectively activated and expanded through the Phyduxon-T manufacturing process *in vitro*. Cells from patients with OC were harvested on day 0 (fresh PBMCs) and day 12 (Phyduxon-T) and stained with monoclonal antibodies to acquire T cell phenotype via flow cytometry. **a** We evaluated the changes in CD4 and CD8 on T cells, (**b**) the expression levels of CCR7^+^CD45RO^+^(central memory) and CCR7^-^CD45RO^+^(effector memory) on CD14^−^CD19^−^CD56^−^TCRγδ^−^CD4^−^CD45^+^TCRαβ^+^CD8^+^ cells, (**c**, **d**) the expression levels of CD25 and CD69 on CD14^−^CD19^−^CD56^−^TCRγδ^−^CD4^−^CD45^+^CD3^+^TCRαβ^+^CD8^+^cells, (**e**, **f**) the expression level of IFN-γ on CD14^−^CD19^−^CD56^-^CD3^+^CD8^+^4-1BB^+^ cells. The results shown in (**c**) and (**e**) are representative of six independent experiments. Data are presented as means ± SD of six independent experiments. Differences between groups were analyzed using the nonparametric Mann-Whitney test. *p *values:*p < 0.05,**p < 0.01

After the 12-day culture, we evaluated CD4 and CD8 T cell changes, observing CD8 T cell expansion compared to fresh PBMCs (CD4: *p* = 0.0087, CD8: *p* = 0.0043) (Fig. 4a). Additionally, we examined the differentiation phenotype of CD8 T cells, revealing an increase in CCR7^−^CD45RO^+^ effector memory CD8 T cells (*p* = 0.0022) and CCR7^+^CD45RO^+^ central memory CD8 T cells (*p* = 0.0087) (Fig. 4b and Fig. S3a). Furthermore, we observed a significant increase in the expression levels of T cell activation markers, such as CD25 (*p* = 0.0022) and CD69 (*p* = 0.0411), on CD8 T cells (Fig. 4c and d and Fig. S3b). IFN-γ production by 4-1BB^+^ CD8 T cells, a phenotype known as TAA-specific CD8 T cells, was significantly increased in vitro (*p* = 0.0043) (Fig. 4e and f, and Fig. S3c). These findings demonstrate that the Phyduxon-T manufacturing platform enables the expansion of a small amount of TAA-specific CD8 T cells derived from the peripheral blood.

### Expanding AIM^+^CD8 T cells promotes T cell-mediated cytotoxicity against primary tumors

The Phyduxon-T immune composition, comprising Phyduxon and TAA-specific T cells, demonstrated significant overall cytotoxicity against primary cancer. OC cells exposed to Phyduxon-T exhibited cytotoxicity of 24.25%, evidenced by caspase/granzyme B, whereas OC cells alone exhibited cytotoxicity of 9.71% (*p* = 0.0022) (Fig. S4). These findings demonstrate that Phyduxon-T exhibits antitumor activity in vitro.

**Fig. 5:**
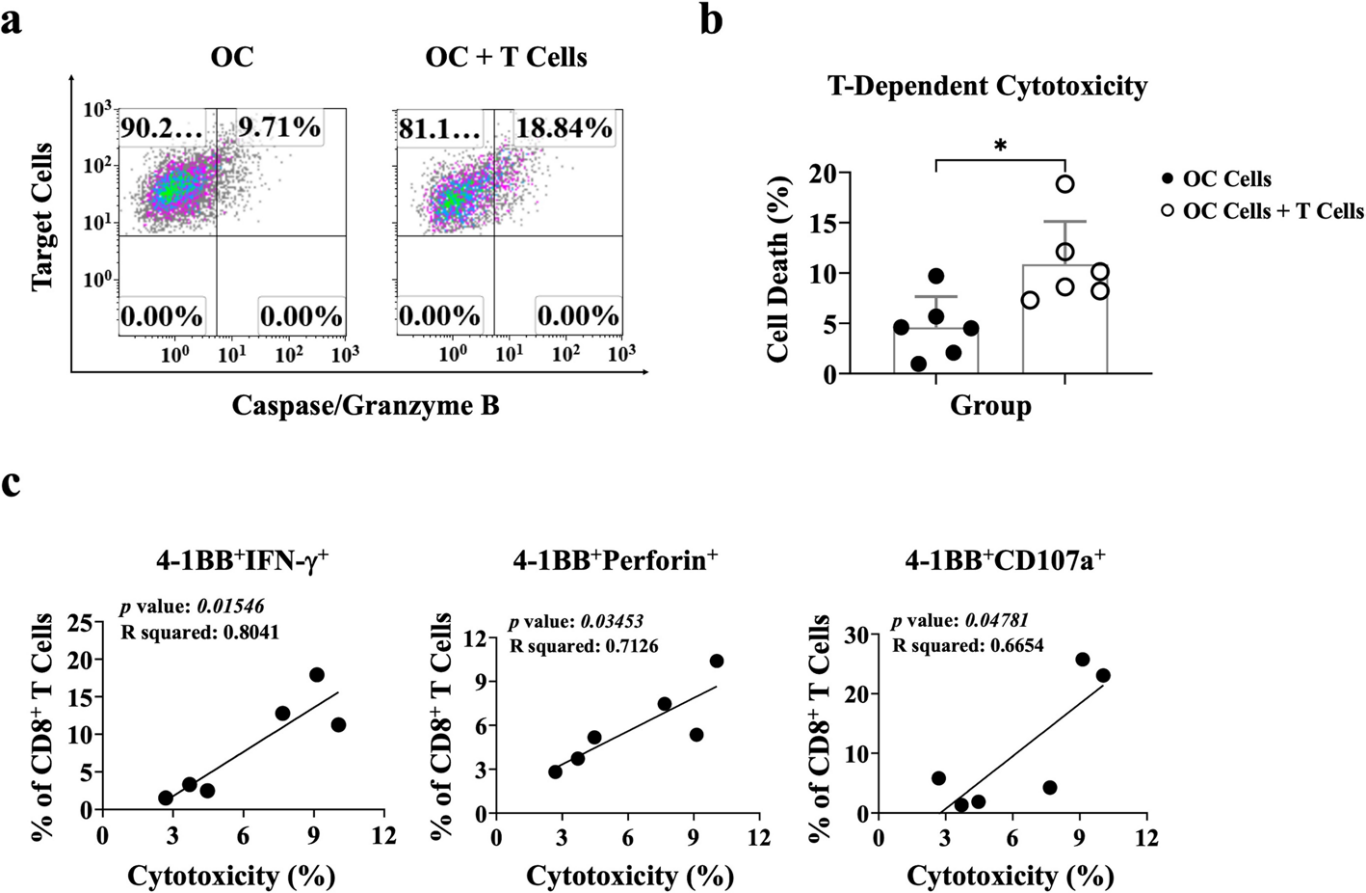
AIM^+^ CD8 T cells exhibited T cell-dependent cytotoxicity against primary epithelial OC. (**a**, **b**) OC cells were cocultured with T cells, and the TFL-4^+^caspase/granzyme B^+^ pattern was analyzed through flow cytometry to quantify apoptosis. **c** The correlation between T cell-dependent cytotoxicity and the percentage of 4-1BB^+^IFN-γ^+^, 4-1BB^+^perforin^+^and 4-1BB^+^CD107a^+^on CD8 T cells was determined. The results shown in (**a**) are representative of six independent experiments. Data are presented as means ± SD of six independent experiments. Differences between groups were analyzed using the nonparametric Mann-Whitney test. *p *values:*p < 0.05

Phyduxon-T exhibited slightly higher cytotoxicity against primary OC cells compared to Phyduxon (Fig. 3d and Fig. S4), indicating that the T cells within Phyduxon-T may be responsible for executing T cell-dependent cytotoxicity. To confirm this, we isolated approximately 90% of CD3 T cells from Phyduxon-T and cocultured them with OC cells to assess their tumor specificity (Fig. S2b). Initially, we cocultured T cells and Phyduxon with K562 cells to assess innate-cell-like cytotoxicity, revealing that Phyduxon, but not T cells (*p* = 0.1032), exhibited NK-mediated innate-cell-like killing activity against K562 cells (*p* = 0.0002) (Fig. S5). Conversely, the isolated T cells demonstrated T cell-dependent cytotoxicity (rate of apoptosis in OC cells: 18.84%), approximately one-fold higher (*p* = 0.0152) than that observed in OC cells alone (9.71%; Fig. 5a and b). Furthermore, we observed strong correlations between the levels of the production of IFN-γ, perforin, and CD107a by 4-1BB^+^ CD8 T cells and their ability to exert T cell-dependent cytotoxicity by inducing apoptosis in OC cells (Fig. 5c). These findings indicate that T cells expanded through the Phyduxon-T manufacturing process exhibit T cell-dependent tumor-specific cytotoxicity against primary tumors, providing evidence for the capability of the Phyduxon-T manufacturing process to expand TAA-specific T cells and combat tumors in vitro.

## Discussion

**Fig. 6:**
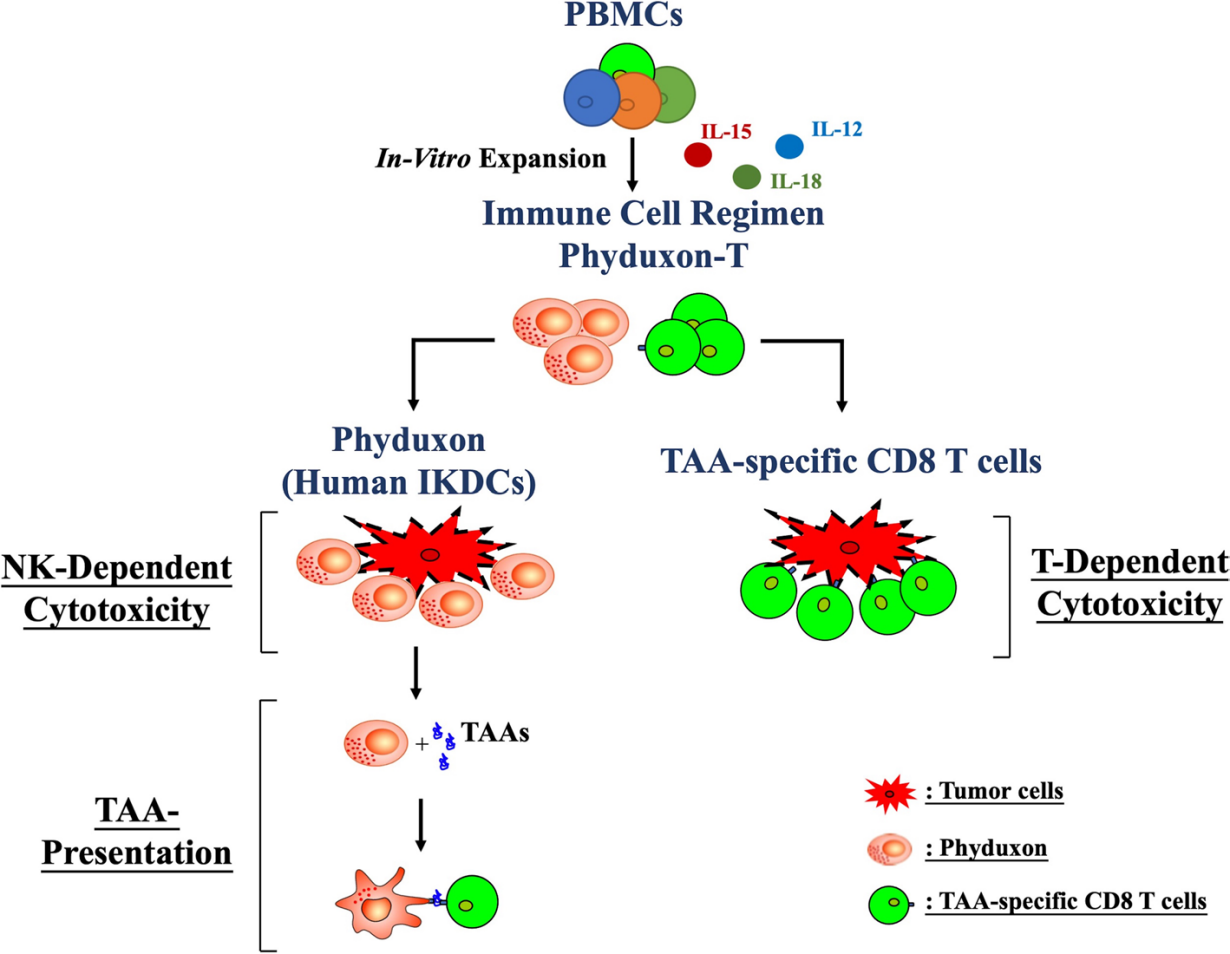
Phyduxon-T, as an immune cell-based regimen, exhibits the characteristics of NK cells, DCs, and TAA-specific CD8 T cells. Phyduxon-T is a novel immune cell-based regimen consisting of the human IKDC counterpart, Phyduxon, and TAA-specific CD8 T cells. Phyduxon demonstrates the ability to eliminate tumor cells and present TAAs, thereby inducing TAA-specific CD8 T-cell responses. The manufacturing protocol of Phyduxon-T facilitates the expansion of TAA-specific T cells, enhancing their potential for tumor destruction. In summary, Phyduxon-T exhibits the characteristics of NK cells, DCs, and T cells, mimicking immunosurveillance and providing a new strategy for adoptive cell transfer therapy

In this study, we developed a manufacturing process to generate a novel adoptive cell transfer regimen called Phyduxon-T. This regimen involves culturing PBMCs with hIL-15, hIL-12, and hIL-18 for 12 days to generate a human IKDC counterpart, Phyduxon, and TAA-specific CD8 T cells in vitro. Phyduxon exhibited NK-dependent killing activity and APC function, while the TAA-specific CD8 T cells were responsible for T cell-dependent cytotoxicity toward OC cells (Fig. 6).

Mouse IKDC was first discovered in 2006 and defined as NK1.1^+^B220^+^CD11c^int^ [7, 8]. Mouse IKDCs secrete IFN-γ and mediate tumoricidal activity in a TRAIL-dependent manner [8] and also secrete IL-12 to upregulate the expression of MHC-II and costimulatory molecules, thereby acquiring APC function [7, 29]. Adoptively transferring this particular immune subset, exhibiting the phenotype and properties of both NK cells and DCs, results in antitumor activity in vivo [9]. The human counterpart of IKDC expresses CD56 and HLA-DR and can be expanded in vitro in response to cytokines such as IL-15, IL-12, and IFN-γ [10–12]; this expanded IKDC exhibits the functional activities of both NK cells and DCs [30]. In a previous study, we investigated using IL-15 combined with IL-12 or IL-18 by varying the timing and duration of exposure to establish a new platform for manufacturing human IKDCs [13]. The initial phase of cell preparation involved cell depletion to eliminate unwanted cells before manufacturing [11–13]. In this study, we manufactured Phyduxon-T to generate a novel human IKDC counterpart, Phyduxon, by culturing PBMCs with hIL-15, hIL-12, and hIL-18 for 12 days, eliminating the need for the modification of the initial cells (Figs. 1 and 2). This innovative manufacturing protocol proves to be more practical for producing human IKDC products in clinical settings.

Several methods have been proposed to generate TAA-specific T cells targeting tumor epitopes. First, the adoptive transfer of DCs combined with cytokine-induced killer (DC-CIK) cells can induce TAA-specific T cells in vivo [31, 32]. However, ensuring quality control during preparation poses challenges, complicating the manufacturing process. Second, neoantigen prediction can provide extensive information on somatic mutations in tumor cells, generating a wide range of TAA epitope candidates [33]. Nonetheless, validating the vast number of TAA epitopes is challenging and demands considerable time and effort, as only a limited subset can provoke effective antitumor responses [33, 34]. Studies using murine and human models have demonstrated that IKDC can induce downstream CD8 T-cell proliferation after eliminating tumor cells [9, 35]. Our data also revealed that Phyduxon eliminated tumors and processed tumor antigens, triggering the expansion of downstream 4-1BB^+^IFN-γ^+^ CD8 T cells (Fig. 3); this indicates the emergence of tumor-specific CD8 T cells [36, 37]. This approach significantly reduces the complexity of manufacturing DC-CIK cells. Furthermore, this natural T-cell induction protocol simplifies the currently complex process of validating neoantigen epitopes for selecting TAA-specific T cells.

OC has a low mutation burden, resulting in a low frequency of neoantigen-specific T cells and poor clinical outcomes [38]. This study used proinflammatory cytokines, including IL-15, IL-12, and IL-18, to activate T cells and expand memory CD8 T cells. We further discovered that rare IFN-γ-producing 4-1BB^+^ CD8 T cells from peripheral blood, representing TAA-specific T cells, can be expanded through the Phyduxon-T manufacturing process; these cells exhibited T cell-dependent cytotoxicity (Figs. 4 and 5). These findings are consistent with studies reporting that overnight incubation with common γ-chain cytokines, such as IL-7 and IL-15, can upregulate the expression of 4-1BB in T cells [37]. Furthermore, most TIL repertoires comprise 4-1BB^+^TIL cells secreting IFN-γ, which plays a crucial role in the immune response to tumor cells [37, 39]. Our Phyduxon-T manufacturing protocol enabled the expansion of IFN-γ-producing 4-1BB^+^CD8 T cells, perforin, and CD107a by using peripheral blood samples from patients with OC (Fig. 5c). This approach holds promise for enhancing clinical outcomes in patients with OC or other cancers.

Studies have demonstrated a phenomenon known as bystander T-cell activation, characterized by the expansion and activation of T cells with innate-cell-like activities in response to microbial and viral infections or tumors. These innate-cell-like cells exhibit innate-like cell cytotoxicity independently of the TCR [40, 41]. For instance, IL-15 alone or a combination of IL-12 and IL-18 has been shown to induce IFN-γ production and upregulate CD69 expression [42]. In acute hepatitis A, these innate-cell-like cells are activated through bystander cytokines within a short period to exhibit NK cell-like cytotoxicity independently of the TCR but through NKG2D and its ligand [43, 44]. However, T cells from Phyduxon-T exhibited T cell-dependent cytotoxicity against primary OC cells instead of innate-like cell cytotoxicity against K562 cells (Fig. 5a and b and Fig. S5). This discrepancy may be attributed to the fact that innate-like cell cytotoxicity is typically induced within a short period of approximately 48 h and may exhibit NK cell-like activity during the initial induction phase. A previous study showed that the extended cultivation period triggered the production of IFN-γ by IL-7 and IL-15 overnight-treated 4-1BB^+^ T cells after engaging with tumors, resulting in T-dependent killing [37]. Our Phyduxon-T manufacturing protocol entails a long cultivation period in the presence of a unique cytokine combination to induce a T-mediated immune response rather than an innate-like immune response.

This study demonstrated that Phyduxon-T has the potential to initiate TAA-specific CD8 T cell responses and execute T-dependent tumoricidal activities *in vitro.* However, limitations arise from the suppressive tumor microenvironments, hindering the migration and infiltration of TAA-specific CD8 T cells and their corresponding cytotoxicity against cancer [45]. Therefore, a future therapeutic strategy combining immune checkpoint inhibitors to alleviate the immune suppression of the tumor microenvironment, along with ACT, could be a promising approach to treating solid tumors [46].

## Conclusions

In conclusion, our study presents the successful development of Phyduxon-T, a novel immune cell regimen that integrates NK, DC, and T cell functions. This regimen effectively selects and expands TAA-specific T cells from the peripheral blood of patients. By bridging innate and adaptive immunity, Phyduxon-T initiates immunosurveillance, effectively overcoming tumor immune escape, a frequent obstacle in solid tumors. This ultimately facilitates the delivery of potent anti-tumor immune responses.

### Supplementary Information

**Supplementary Material 1.**

## Data Availability

The datasets used and/or analyzed during the current study are available from the corresponding author upon reasonable request.

## Abbreviations

NK cells: Natural killer cells
MHC: Major histocompatibility complex
TAAs: Tumor-associated antigens
APCs: Antigen-presenting cells
DCs: Dendritic cells
CAR-T cells: Chimeric antigen receptor T cells
TCR-T cells: T-cell receptor (TCR)-engineered T cells
IFN: Interferon
IKDCs: Interferon-producing killer dendritic cells
OC: Ovarian cancer
PBMCs: Peripheral blood mononuclear cells
MCDB: Molecular Cellular Developmental Biology
IMDM: Iscove’s Modified Dulbecco's Medium
PBS: Phosphate-buffered saline
MACS: Magnetic-activated cell sorting
SA: Streptavidin
IL: Interleukin
HPL: Human platelet lysate
CFSE: Carboxyfluorescein succinimidyl ester
Lin: Lineage
AIMs: Activation-induced markers
DC-CIK cells: DCs combined with cytokine-induced killer cells
