# Supplementary material for: Characterization of tumoricidal activities mediated by a novel immune cell regimen composing interferon-producing killer dendritic cells and tumor-specific cytotoxic T lymphocytes
Source: BMC Cancer. 2024 Mar 28;24:395. doi: 10.1186/s12885-024-12101-3 (PMC10979599; doi:10.1186/s12885-024-12101-3)
Supplement: Supplementary file 1 — Supplementary Material 1. [file 12885_2024_12101_MOESM1_ESM.pdf]

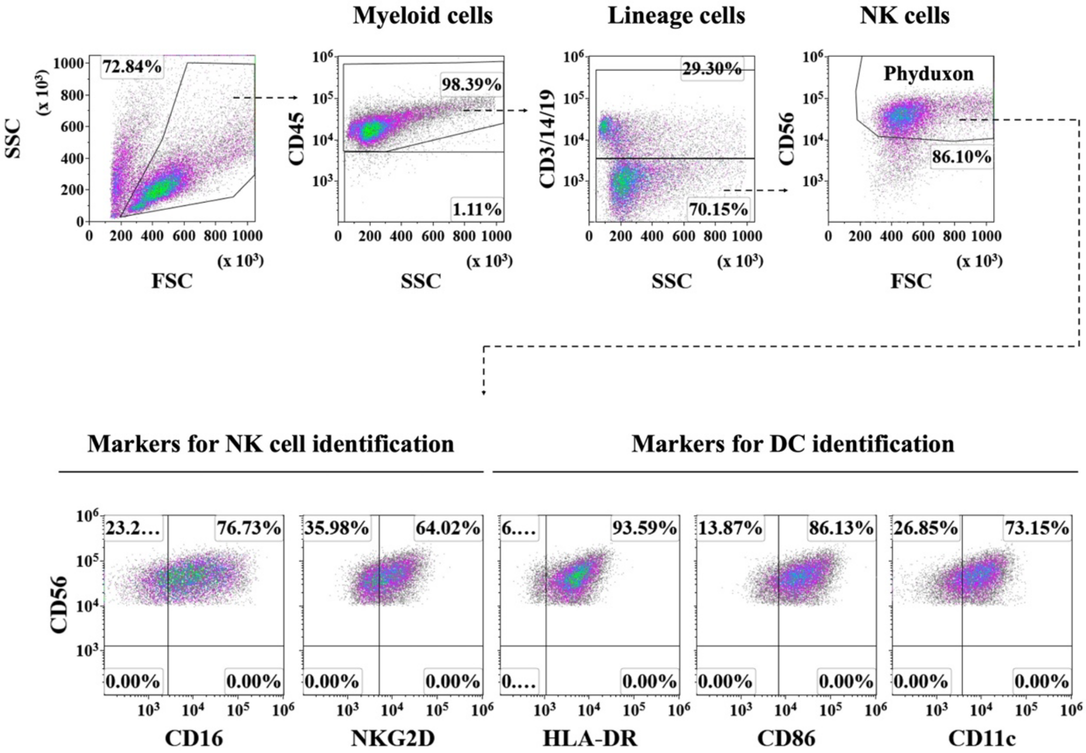

Fig. S1. Pedigree of Phyduxon analysis.

Phyduxon-T was harvested on day 12 and labeled with monoclonal antibodies for the analysis of Phyduxon. The entire cell population was gated on the basis of CD45 expression to identify myeloid cells. Phyduxon was selected from the population of $CD3^{-}CD14^{-}CD19^{-}$ cells (Lin^{-}) and $CD56^{+}$ cells. Finally, the expression levels of CD16, NKG2D, HLA-DR, CD86, and CD11c in Phyduxon were evaluated.

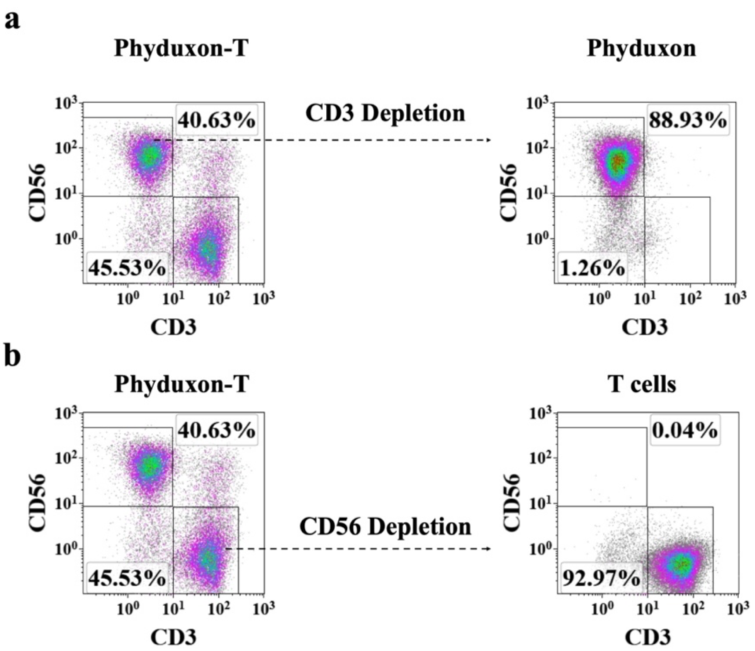

Fig. S2. Isolation of pure Phyduxon and T cells through MACS cell separation.

Phyduxon-T was harvested on day 12 and labeled with biotinylated anti-CD3 and biotinylated anti-CD56 for isolating **(a)** Phyduxon and **(b)** T cells, respectively. The biotin-conjugated cells were incubated with SA microbeads and passed through the LD columns to collect the eluted fraction (untouched).

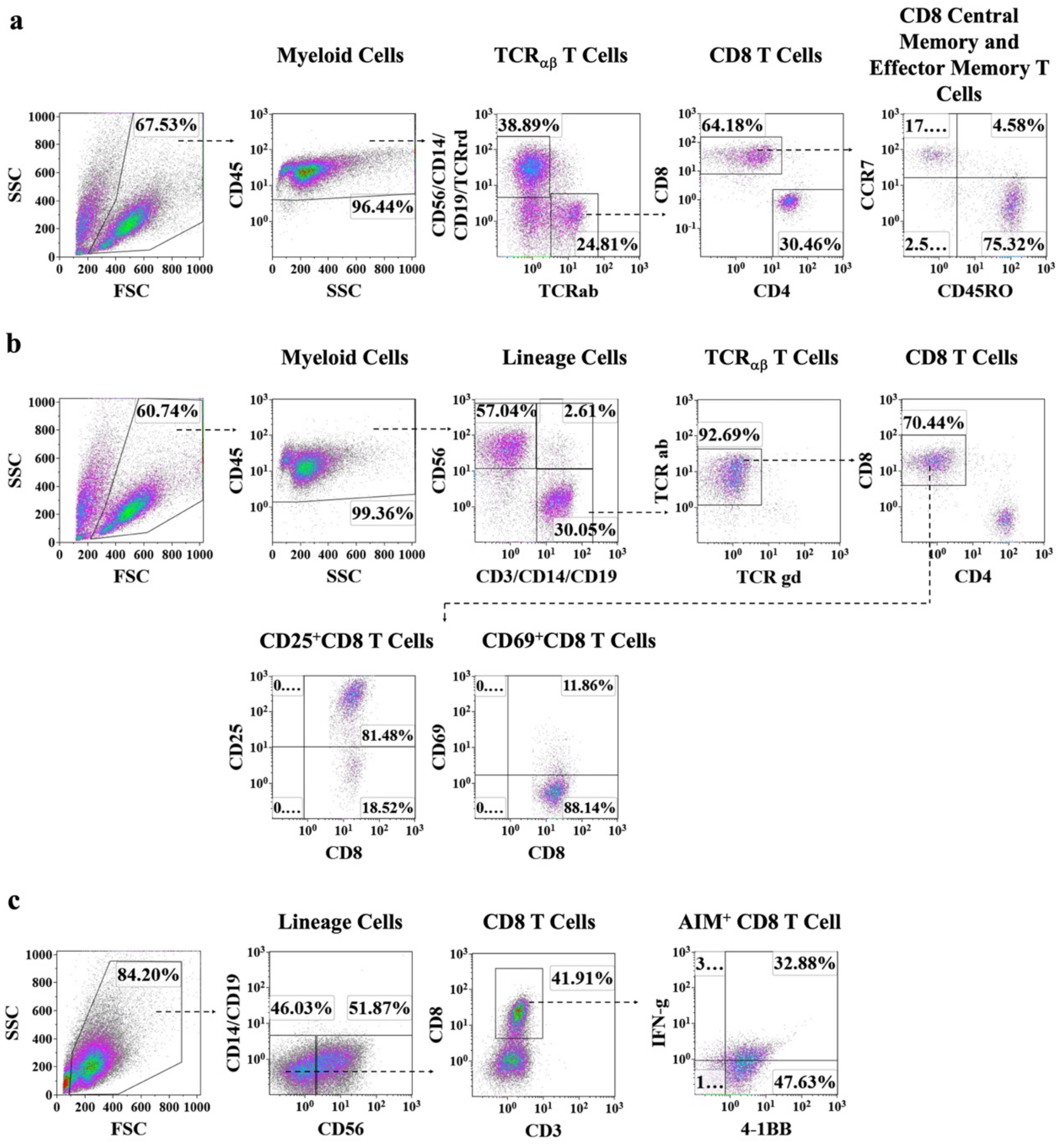

Fig. S3. Pedigree of T-cell analysis.

(a) Phyduxon-T was harvested on day 12 and labeled with monoclonal antibodies for T-cell determination. The entire cell population was gated on the basis of CD45 expression to identify myeloid cells. TCRαβ T cells were selected from the population of $CD14^{-}CD19^{-}CD56^{-}TCR\gamma\delta^{-}$ and $TCR\alpha\beta^{+}$ cells. CD8 T cells were selected from the population of $CD4^{-}CD8^{+}$ cells. Finally, the expression levels of CD45RO and CCR7 in $CD45^{+}CD14^{-}CD19^{-}CD56^{-}TCR\gamma\delta^{-}TCR\alpha\beta^{+}CD4^{-}CD8^{+}$ cells were evaluated for

assessing effector ($CD45RO^{+}CCR7^{-}$) and central memory ($CD45RO^{+}CCR7^{+}$) CD8 T cells. **(b)** The entire cell population was gated on the basis of CD45 expression to identify myeloid cells. Lineage cells were selected from the population of $CD3^{+}CD14^{+}CD19^{+}CD56^{-}$ cells. Next, we correlated $CD45^{+}CD3^{+}CD14^{+}CD19^{+}CD56^{-}$ cells with TCRγδ and TCRαβ to define TCRαβ T cells as $CD45^{+}CD14^{-}CD19^{-}CD3^{+}CD56^{-}TCR\gamma\delta^{-}TCR\alpha\beta^{+}$. Finally, CD8 T cells were selected from the population of $CD4^{-}CD8^{+}$ cells; the expression levels of CD25 and CD69 on CD8 T cells were evaluated. **(c)** Lineage cells were selected from the population of $CD14^{-}CD19^{-}CD56^{-}$ cells. We further correlated $CD14^{-}CD19^{-}CD56^{-}$cell with CD3 and CD8 to define CD8 T cells as $CD14^{-}CD19^{-}CD56^{-}CD3^{+}CD8^{+}$ cells. Finally, the expression levels of 4-1BB and IFN-γ on $CD14^{-}CD19^{-}CD56^{-}CD3^{+}CD8^{+}$ cells were evaluated to assess AIM^{+} CD8 T cells.

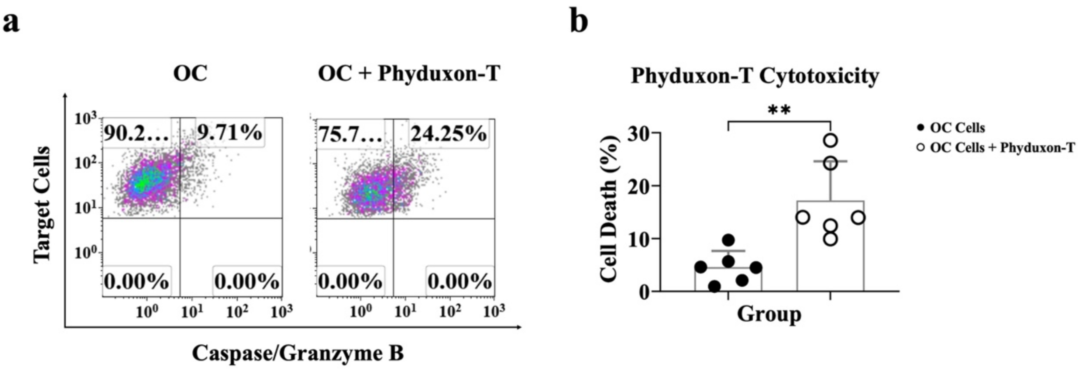

Fig. S4. Phyduxon-T exhibited tumoricidal activity against primary ovarian cancer.

OC cells were cocultured with Phyduxon-T. The TFL-4^{+}caspase/granzyme B^{+} pattern was analyzed through flow cytometry to quantify apoptosis. The results shown in **(a)** are representative of six independent experiments. Data are presented as means ± SD of six independent experiments. Differences between groups were analyzed using the nonparametric Mann-Whitney test. p values: * $p < 0.05$, ** $p < 0.01$.

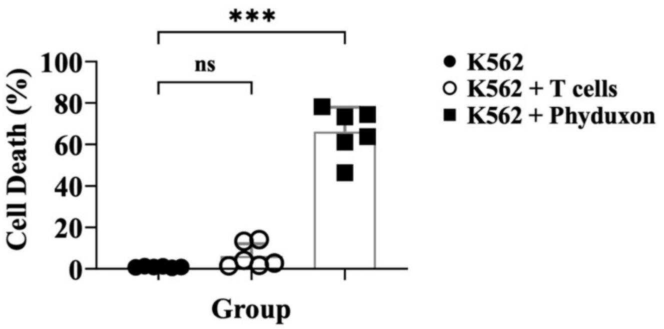

Fig. S5. Evaluation of innate-cell-like cytotoxicity in Phyduxon and T cells.

K562 cells were cocultured with T cells and Phyduxon. The TFL-4^{+}caspase/granzyme B^{+} pattern was analyzed through flow cytometry to quantify apoptosis. All experiments included six independent experiments. Data are presented as means ± SD of six independent experiments. Differences between groups were analyzed using the nonparametric Mann-Whitney test. *p* values: * $p < 0.05$, ** $p < 0.01$; ns, not significant.

Table S1. Patient Demographics

Patient (n=6)	Age	Historical Subtypes	Stage
NTUH-001	55	Low-grade serous carcinoma (LGSC)	IIIC
NTUH-002	62	Clear cell carcinoma	IA
NTUH-003	51	Clear cell carcinoma	IIB
NTUH-004	56	High-grade serous carcinoma (HGSC)	IIIC
NTUH-005	61	High-grade serous carcinoma (HGSC)	IVB
NTUH-007	74	High-grade serous carcinoma (HGSC)	IIIB

Table S2. Reagents

Reagents	Catalog	Manufacturer	City	State	Country
Cell Lines and Primary Tumor Cell Isolation					
Iscove’s Modified Dulbecco's Medium (IMDM)	12440053	Gibco	Waltham	MA	USA
Fetal bovine serum (FBS)	10437028	Gibco	Waltham	MA	USA
Collagenase type IV	C5138	Sigma	Burlington	MA	USA
DNase I	D5025	Sigma	Burlington	MA	USA
Hyaluronidase	H3757	Sigma	Burlington	MA	USA
Medium 199	11150059	Gibco	Waltham	MA	USA
MCDB 105 Medium	117-500	Cell Applications	San Diego	CA	USA
Phosphate-buffered saline (PBS)	21-040-CV	Corning	Corning	NY	USA
Ficoll-Hypaque	17-5442-03	Cytiva	Marlborough	MA	USA
PBMC Isolation and Cell Enrichment					
Streptavidin microbeads	130-048-101	Miltenyi Biotec	Bergisch Gladbach	Rheinisch-Bergischer Kreis	Germany

LD columns	130-042-901	Miltenyi Biotec	Bergisch Gladbach	Rheinisch-Bergischer Kreis	Germany
Preparation of Phyduxon-T					
AIM-V medium	0870112DK	Gibco	Waltham	MA	USA
Human platelet lysate (HPL)	HPCFDCRL50	AventaCell	Atlanta	GA	USA
Human interleukin-15 (hIL-15)	247-GMP-025	R&D	Minneapolis	MN	USA
Human interleukin-12 (hIL-12)	170-076-174	Miltenyi Biotec	Bergisch Gladbach	Rheinisch-Bergischer Kreis	Germany
Human interleukin-18 (hIL-18)	592106	BioLegend	San Diego	CA	USA
Flow Cytometry					
Cell Stimulation Cocktail	00-4970-03	eBioscience	San Diego	CA	USA
BD Cytofix/Cytoperm Plus Fixation/Permeabiliz	554715	BD Biosciences	Franklin Lakes	NJ	USA

ation Solution Kit with BD GolgiStop					
Cytotoxic Assay					
PanToxiLux kit	PTL8028	OncoImmunin	Gaithersburg	MD	USA
APC Activity Assay					
CellTrace CFSE cell proliferation kit	C34554	Invitrogen	Carlsbad	CA	USA
Human interleukin-2 (hIL-2)	791908	BioLegend	San Diego	CA	USA
Human interleukin-15 (hIL-15)	570308	BioLegend	San Diego	CA	USA
CellTrace Violet cell proliferation kit	C34557	Invitrogen	Carlsbad	CA	USA

Table S3. Antibodies for Cell Enrichment

Antibodies	Isotype	Clone	Catalog	Manufacturer	City	State	Country
Biotinylated anti-CD25	Mouse IgG1, κ	BC96	302624	BioLegend	San Diego	CA	USA
Biotinylated anti-CD56	Mouse IgG1, κ	HCD56	318320	BioLegend	San Diego	CA	USA
Biotinylated anti-CD4	Mouse IgG2b, κ	OKT4	317406	BioLegend	San Diego	CA	USA
Biotinylated anti-CD11c	Mouse IgG1, κ	3.9	301612	BioLegend	San Diego	CA	USA
Biotinylated anti-CD14	Mouse IgG1, κ	HCD14	325624	BioLegend	San Diego	CA	USA
Biotinylated anti-CD19	Mouse IgG1, κ	HIB19	302204	BioLegend	San Diego	CA	USA
Biotinylated anti-CD3	Mouse IgG1, κ	UCHT1	300404	BioLegend	San Diego	CA	USA

Table S4. Antibodies for Phyduxon and T cell immune profiling

Antibodies	Isotype	Clone	Catalog	Manufacturer	City	State	Country
Phyduxon							
NKG2D-PE	Mouse IgG1	ON72	A08934	Beckman Coulter	Brea	CA	USA
CD45-ECD	Mouse IgG1	J33	A07784	Beckman Coulter	Brea	CA	USA
CD56-APC-Alexa Fluor 700	Mouse IgG1	N901	B10822	Beckman Coulter	Brea	CA	USA
CD3-APC-Alexa Fluor 750	Mouse IgG1	UCHT1	A66329	Beckman Coulter	Brea	CA	USA
CD14-APC-Alexa Fluor 750	Mouse IgG2a	RMO52	A86052	Beckman Coulter	Brea	CA	USA
CD19-APC-Alexa Fluor 750	Mouse IgG1	J3-119	A78838	Beckman Coulter	Brea	CA	USA
HLA-DR-Krome orange	Mouse IgG1	Immu-357	B00070	Beckman Coulter	Brea	CA	USA
CD86-Alexa Fluor 488	Mouse IgG2b, κ	IT2.2	305414	BioLegend	San Diego	CA	USA
CD16-PE-Cy7	Mouse IgG1	3G8	6607118	Beckman Coulter	Brea	CA	USA
CD11c-APC	Mouse IgG1	3.9	301614	BioLegend	San Diego	CA	USA

T cells							
CD45-ECD	Mouse IgG1	J33	A07784	Beckman Coulter	Brea	CA	USA
CD56-APC-Alexa Fluor 700	Mouse IgG1	N901	B10822	Beckman Coulter	Brea	CA	USA
CD3-APC-Alexa Fluor 750	Mouse IgG1	UCHT1	A66329	Beckman Coulter	Brea	CA	USA
CD14-APC-Alexa Fluor 750	Mouse IgG2a	RMO52	A86052	Beckman Coulter	Brea	CA	USA
CD19-APC-Alexa Fluor 750	Mouse IgG1	J3-119	A78838	Beckman Coulter	Brea	CA	USA
CD8-Krome Orange	Mouse IgG1	B9.11	B00067	Beckman Coulter	Brea	CA	USA
TCRαβ-FITC	Mouse IgG1, κ	IP26	306706	BioLegend	San Diego	CA	USA
4-1BB-PE	Mouse IgG1, κ	4B4-1	309804	BioLegend	San Diego	CA	USA
CD25-PE	Mouse IgG1, κ	BC96	302606	BioLegend	San Diego	CA	USA
CD69- Pacific Blue	Mouse IgG1, κ	FN50	310920	BioLegend	San Diego	CA	USA
CD4-PE-Cy7	Mouse IgG1	SFCI12T4D11	6607101	Beckman Coulter	Brea	CA	USA
CD45RO-APC	Mouse IgG2a, κ	UCHL1	304210	BioLegend	San Diego	CA	USA

CCR7-PE-Cy7	Mouse IgG2a, κ	G043H7	353226	BioLegend	San Diego	CA	USA
TCRγδ-APC	Mouse IgG1, κ	B1	331212	BioLegend	San Diego	CA	USA

Table S5. Antibodies for cytotoxic molecule immune profiling

Antibodies	Isotype	Clone	Catalog	Manufacturer	City	State	Country
Cytotoxic Molecules							
CD107a-PE/Dazzle™ 594	Mouse IgG1, κ	H4A3	328646	BioLegend	San Diego	CA	USA
CD8-Krome Orange	Mouse IgG1	B9.11	B00067	Beckman Coulter	Brea	CA	USA
4-1BB-PE	Mouse IgG1, κ	4B4-1	309804	BioLegend	San Diego	CA	USA
Perforin-FITC	Mouse IgG2b, κ	dG9	308104	BioLegend	San Diego	CA	USA
IFN-γ- PerCP/Cy5.5	Mouse IgG1, κ	4S.B3	502526	BioLegend	San Diego	CA	USA
